# Prevalence and multi-level factors associated with acute malnutrition among children aged 6–59 months from war affected communities of Tigray, Northern Ethiopia, 2021: a cross-sectional study

**DOI:** 10.1186/s13031-023-00508-x

**Published:** 2023-03-18

**Authors:** Gebretsadkan Gebremedhin Gebretsadik, Mahlet Abraha, Tedros Bereket, Ferehiwot Hailemariam, Freweini Gebrearegay, Tigist Hagos, Mizan Assefa, Kidanemaryam Berhe, Hadush Gebregziabher, Amaha Kahsay Adhanu, Mekonnen Haileselassie, Mulugeta Gebregziabher, Afework Mulugeta

**Affiliations:** 1 Department of Nutrition and Dietetics, School of Public Health, College of Health Sciences, Mekelle University, Mekelle, Tigray Ethiopia; 2 Department of Environmental Health and Behavioral Sciences, School of Public Health, College of Health Sciences, Mekelle University, Mekelle, Tigray Ethiopia; 3 Department of Reproductive Health, School of Public Health, College of Health Sciences, Mekelle University, Mekelle, Tigray Ethiopia; 4 Tigray Health Bureau, Mekelle, Tigray Ethiopia; 5 Department of Public Health Sciences, Medical University of South Carolina, Charleston, SC 29425 USA

**Keywords:** War, Tigray, Acute malnutrition, Children under-five, Associated factors, Multilevel

## Abstract

**Background:**

Armed conflicts greatly affect the health, nutrition, and food security of conflict affected settings particularly children. However, no empirical data exist regarding context specific factors contributing towards acute malnutrition in the war-torn Tigray, Ethiopia. Thus, this study aimed to identify individual and community level factors associated with acute malnutrition among children aged 6–59 months from armed conflict affected settings of Tigray, Ethiopia.

**Methods:**

A community based cross-sectional study was conducted among 3,614 children aged 6–59 months in Tigray, from July 15 to Aug 15, 2021. Study participants were selected using a two-stage random sampling method. A structured questionnaire was used to collect data by interviewing mothers/caregivers. Mid upper arm circumference (MUAC) measurements were taken from upper left arm of the children using MUAC tapes. Multivariable multilevel logistic regression analysis was used to determine factors associated with acute malnutrition. Adjusted Odds ratio (AOR) with 95% CI were estimated to describe the strength of associations at *p* < 0.05.

**Results:**

More than half (52.5%) of the sampled children were males in sex. Immediately after the first nine months into the conflict, the prevalence of severe, moderate, and global acute malnutrition was very high (5.1%, 21.8%, and 26.9%, respectively) in Tigray. The lowest and highest burden of child acute malnutrition was reported from Mekelle zone (13.3%) and Southeastern zone (36.7%), respectively. Individual-level factors such as older child age (AOR = 0.13, 95% CI: 0.10, 0.18), female child sex (AOR = 1.24, 95% CI 1.05, 1.480.95), Vitamin-A supplementation (AOR = 1.3, 95% CI: 1.05, 1.65), and history of diarrhea (AOR = 1.22, 95%CI: 1.02, 1.53) and community-level factors like unimproved drinking water source (AOR = 1.31, 95%CI: 1.08, 1.58), unimproved toilet facility (AOR = 1.24, 95% CI: 1.01, 1.52), and severe food insecurity (AOR = 1.55, 95% CI: 1.16. 2.07) were significantly associated with childhood acute malnutrition.

**Conclusions:**

The burden of acute malnutrition is a severe public health problem in Tigray. To prevent the untimely suffering and death of children, regular nutrition screening, speedy, and appropriate referral of all malnourished children to nutritional services and large-scale humanitarian assistance including access to food; nutrition supplies; water, sanitation and hygiene supplies; and health care in a timely manner are required. In the prevailing armed conflict, these have been very difficult to achieve. Thus, immediate international intervention is needed.

**Supplementary Information:**

The online version contains supplementary material available at 10.1186/s13031-023-00508-x.

## Background

The burden of disease and the mortality levels experienced by the populations of countries with armed conflicts are extraordinarily high. Armed conflicts have devastating effects on the delivery of and access to health services not only during the period of conflicts but also in the aftermath of the conflicts. Evidences have shown that children are disproportionately affected in armed conflict settings [1, 2]. About 60 percent of all chronically food-insecure and malnourished people globally, including 75 percent of all stunted children, live in conflict-affected countries [3].

Similarly, studies from Sub-Saharan Africa (SSA) reported high rates of conflict associated acute malnutrition and mortality among under-five children [4–11]. Moreover, armed conflicts increased death rates by up to 24 times [12] and intensified household food insecurity, and deprival of basic needs including food, shelter, safe water, and adequate sanitary and appropriate equipment to maintain hygiene [13]. In Tigray, Global Acute Malnutrition (GAM) among 6–59 months old children increased almost three times from 10% in 2019 (prewar) to 28% in 2021 after eight months into the war [14]. Moreover, Severe Acute Malnutrition (SAM) increased up to six times from 1% in 2019 (pre-war) to 6% in 2021 after eight months of the onset of the war [14].

Despite the severity of the health consequences of the armed conflict, we have a very limited knowledge on the context specific factors contributing to the poor nutrition situation of the 6–59 months old children under five children from the armed conflict affected settings of Tigray. In the absence of well-functioning health system and health information system in Tigray, most stakeholders are obliged to rely on numerous media/press reports of eyewitness and facility reports making determinant factors of acute malnutrition difficult to validate. Thus, more reliable data are needed to identify the context specific factors associated with acute malnutrition among children from the conflict affected communities in Tigray. Knowledge and accurate assessments of the risk factors would raise the possibility of preventing and ameliorating the dire health consequences of poor nutritional status among under five children in the armed conflict affected settings in Tigray.

In this study, we hypothesized that the armed conflict had considerable impact on the nutritional status of 6–59 months old children in Tigray. Thus, the objective of this study was to assess individual and community level factors associated with acute malnutrition among 6–59 months old children immediately after eight months into the armed conflict on Tigray, Northern Ethiopia. The factors were identified using a cross-sectional survey.

## Methods

### Study setting

The study was conducted in Tigray, the northern most part of Ethiopia. Tigray is bordered with Eritrea from the north, Sudan from the west, Afar region from the east and Amhara region from the south. Administratively, Tigray is divided in to seven zones namely Central, Eastern, Mekelle, North western, Southern, South eastern and Western zones and 93 districts. In this study, 52 districts in all the zones except the western zone were included. The western zone was excluded for security concerns. The study was conducted between July 15 and Aug 15, 2021.

### Study design and population

A community based cross-sectional study was conducted in six zones and 52 randomly selected districts of Tigray. Only mothers or caretakers of children 6–59 months of age were included in the study. Inclusion criteria: all households with under one years of age children were included. Then, all children under five in the selected household were measured for nutritional status. Exclusion criteria: Serious illness in children under five.

### Sample size determination

The minimum sample size was estimated using proportional allocation of the minimum sample size for a Tabia (smallest administrative unit) from each of the districts. The sample size was determined based on the burden of acute malnutrition. According to the EDHS 2016, the burden of acute malnutrition was 11% in Tigray [15]. For situations where power and prevalence are known, effective sample size can easily be estimated. For 11% prevalence, 20 subjects are sufficient to reach a power value greater than 80 [16]. Accordingly, 20 households were included from each Tabia and four Tabias were selected from each district. Thus, adding 5% of non-response, the total sample size was calculated to be 4368 [20 subjects per Tabia*4 Tabias per district*52 districts) + (5%*4160)]. However, 754 subjects were excluded from the final analysis for the following reasons; 1) 444 were under the age of 6 months; 2) 308 had no MUAC measurements, and 3) 2 were outliers. Therefore, the findings of this study were based on data from 3614 subjects.

### Sampling techniques

A multistage sampling technique was employed. All the zones except the western zone were included in the study. At the first stage, a total of 52 districts were randomly selected from the 93 districts. In the second stage, four Tabias were randomly selected from each of the 52 districts. Then, 20 households with under one year of age children were randomly selected from the selected tabias using the registration book of the Health Extension Workers (HEWs) as a sampling frame. When the registration book of the HEWs was not available, a new list of the households with under one year of age children was generated and used as a sampling frame to randomly select the study households. However, it must be noted that the districts from the Western zone of Tigray were not included in the sampling frame.

### Data collection tool and data quality control

Data were collected using a pretested and interviewer-administered structured questionnaire. The tool contained items regarding socio-demographic, health and obstetric, childhood illness and vaccination characteristics; water, hygiene and sanitation conditions; and household food insecurity, and anthropometric measurement (MUAC). Experienced HEWs were the data collectors and health and nutrition researchers/experts from Mekelle University and Tigray Health Bureau worked as supervisors of the data collection. MUAC measurements were taken from the upper left arm of the children using MUAC tapes; household food insecurity was measured using the Household Food Insecurity Access Scale (HFIAS), which was answered by the mothers/caregivers of the children. The questionnaire was initially prepared in English and then translated into the local language (Tigrigna) and was then translated back to English for consistency check. Three days training was given for the data collectors and supervisors. Moreover, fieldwork was accompanied by strict follow up and supervision.

### Individual-level factors

These included child age, child sex, Vitamin-A supplementation status, measles vaccine status, deworming, maternal education, paternal education, child had diarrhea in the last nine months, child had fever in the last nine months, child had cough in the last nine months, place of delivery, and antenatal care (ANC) visits.

### Community-level factors

Residence, toilet facility, drinking water source, handwashing facility close to toilet, solid waste disposal, liquid waste disposal, family size, and household food insecurity were considered as community level factors.

### Dependent variable

The dependent variable for this study was child acute malnutrition as measured by MUAC with two categories (“Yes” if MUAC < 12.5 cm and “No” if MUAC ≥ 12.5 cm).

### Data analysis

Data (Additional file 1) were cleaned and analyzed using Statistical package for Social Sciences (SPSS) software version 25. Categorical variables were summarized using percentages. Multilevel binary logistic regression analysis was employed to identify factors significantly associated with the outcome variable. Within the multilevel multivariable logistical regression analysis, Adjusted odds ratios (AOR) with their 95% confidence intervals were computed to measure the fixed effects of individual-level and community-levels factors on the prevalence of child acute malnutrition. During bi-variable logistic regression analysis, we used *p*-value of ≤ 0.2 to screen factors for multivariable logistic regression analysis. In the final multivariable logistic model, four models including an intercept-only model containing no explanatory variables, an individual-only model, a community-only model, and a combined model containing both individual and community-level variables were fitted. This helped to come up with a model where the effect of clustering is controlled and to determine the independent effect of each individual and community level factors on the dependent variable. Associations were declared statistically significant at a *p*-value of < 0.05.

Prior to running the multivariable logistic analysis, multicollinearity among individual and community level variables was checked using Variance Inflation Factor (VIF) cutoff value of 10. From the four fitted models, the one with the lowest value of Akaike information criterion (AIC) and/or Bayesian information criterion (BIC) was selected as the best model to our data. Both AIC and BIC consist of a part that represents model fitness and a part that represent the size and dimensionality of the model as shown in the equation: *IC*= − 2log*f*(*y│Ӫ*)+${\varvec{\lambda}}d$*,* where *IC* stands for information criterion, *f(y│Ӫ)* is the likelihood of the data *y* evaluated using the model parameters, *λ* denotes the penalty weight that differs for AIC and BIC, and *d* represents the size or dimensionality of the model [17].

Variables that showed significant association at *p* ≤ 0.20 in the bivariate analysis were entered to multivariable logistic regression analysis. These included individual level variables like child age, child sex, Vitamin A supplementation, diarrhea in the last nine months, treatment sought for diarrhea, and fever. Community level variables that met this criteria were drinking water source, toilet facility, and HFIAS. Our analysis showed multicollinearity between presence of diarrhea and treatment sought for diarrhea. From the bivariate logistic regression, the *p*-value for treatment sought for diarrhea was higher than the *p*-value for presence of diarrhea, thus, we removed the variable treatment sought for diarrhea.

## Results

### Characteristics of study participants and bivariate analysis of factors with acute malnutrition

In this study, data from 3,614 children with age of 6–59 months is reported. Almost three-fourths (74.1%) of the respondents were females. Slightly higher proportions of male children (52.5%) were included in the study. The average child age (± SD) and household size were 16.8 (± 16.0) months and 4.8 (± 1.90), respectively. Majority (85.3%) of the respondents were not visited by HEWs during the first nine months of the war period prior to the date of the survey. During the first nine months of the armed conflict, about 2,795 (81.1%), 3,540(99.1%), and 3,009 (98.7%) of the 6–59 months old children didn’t receive vitamin A supplementation, measles vaccine, and deworming, respectively (Table 1).

**Table 1:** Characteristics of study participants and bivariate associations of factors with acute malnutrition in 6–59 months age children, Tigray, Northern Ethiopia, 2021 (*n* = 3614)

Individual and community-level variables		Acute malnutrition		Total	*P*-value
		No (%^a^)	Yes (%^a^)	*N* (%^b^)	
Respondent sex	Female	1913 (71.4)	766 (28.6)	2679 (74.1)	0.31
	Male	728 (77.9)	207 (22.1)	935 (25.9)	
Child sex	Male	1309 (74.2)	454 (25.8)	1763 (52.5)	0.034
	Female	1139 (71.3)	458 (28.7)	1597 (47.5)	
Child age in months	6–8	719 (63.5)	414 (36.5)	1133 (31.4)	< 0.001
	9–11	694 (63.2)	404 (36.8)	1098 (30.4)	
	12–23	124 (73.8)	44 (26.2)	168 (4.6)	
	24–59	1104 (90.9)	111 (9.1)	1215 (33.6)	
Received measles vaccine (missing = 43)	No	2585 (73.0)	955 (30.0)	3540 (99.1)	0.29
	Yes	20 (64.5)	11 (35.5)	31 (0.9)	
Received deworming tablets (missing = 565)	No	2161 (71.8)	848 (28.2)	3009 (98.7)	0.43
	Yes	31 (77.5)	9 (22.5)	40 (1.3)	
Number of ANC visits (missing = 221)	None	1007 (72.1)	390 (27.9)	1397 (41.2)	0.92
	1	245 (72.1)	95 (27.9)	340 (10.0)	
	2	282 (73.8)	100 (26.2)	382 (11.3)	
	3	342 (72.9)	127 (27.1)	469 (13.8)	
	4 +	593 (73.7)	212 (26.3)	805 (23.7)	
Place of delivery (missing = 67)	Home	1177 (72.5)	446 (27.5)	1623 (45.8)	0.68
	Health facility	1385 (73.7)	495 (26.3)	1880 (53.0)	
	Others	31 (70.5)	13 (29.5)	44 (1.2)	
Visited by HEWs	No	2250 (72.9)	837 (27.1)	3087 (85.4)	0.53
	Yes	391 (74.2)	136 (25.8)	527 (14.6)	
Supplemented with Vitamin A	No	2001 (71.6)	794 (28.4)	2795 (81.1)	0.003
	Yes	497 (76.5)	153 (23.5)	650 (18.9)	
Diarrhea in the last nine months (missing = 14)	No	1890 (76.1)	594 (33.9)	2484 (69.0)	0.004
	Yes	739 (66.2)	377 (33.8)	1116 (31.0)	
Sought treatment for the diarrhea (*n* = 1116)	No	456 (64.1)	255 (35.9)	711 (63.7)	0.049
	Yes	283 (69.9)	122 (30.1)	405 (36.3)	
Cough in the last nine months (missing = 16)	No	1451 (75.5)	470 (24.5)	1921 (53.4)	0.44
	Yes	1177 (70.2)	500 (29.8)	1677 (46.6)	
Sought treatment for the cough (*n* = 1677)	No	681 (66.5)	343 (33.5)	1024 (61.1)	0.51
	Yes	496 (76.0)	157 (34.0)	653 (38.9)	
Fever in the last nine months (missing = 14)	No	1728 (76.3)	537 (23.7)	2265 (62.9)	0.09
	Yes	901 (67.5)	434 (32.5)	1335 (37.1)	
Sought treatment for the fever (*n* = 1335)	No	513 (63.0)	301 (37.0)	814 (61.0)	0.39
	Yes	388 (74.5)	133 (25.5)	521 (39.0)	
HFIAS	Food secure	397 (79.7)	101 (20.3)	498 (13.8)	< 0.001
	Mildly food insecure	83 (65.9)	43 (34.1)	126 (3.5)	
	Moderately food insecure	670 (78.0)	189 (22.0)	859 (23.8)	
	Severely food insecure	1491 (70.0)	640 (30.0)	2131 (59.0)	
Drinking water source	Improved^c^	1776 (74.8)	597 (25.2)	2373 (65.7)	0.001
	Unimproved	865 (69.7)	376 (30.3)	1241 (34.3)	
Toilet facility	Improved^d^	1197 (75.1)	397 (24.9)	1594 (44.1)	0.015
	Unimproved	1444 (71.5)	576 (28.5)	2020 (55.9)	
Hand washing facility close to latrine	No	2068 (72.4)	789 (27.6)	2857 (84.8)	0.35
	Yes	394 (77.0)	118 (33.0)	512 (15.2)	
Solid waste disposal	Burying/burning	397 (73.0)	147 (27.0)	544 (15.1)	0.53
	Disposing off elsewhere	910 (73.5)	328 (26.5)	1238 (34.3)	
	Disposing off within household yard	1094 (71.2)	442 (28.8)	1536 (42.5)	
	Other	240 (81.1)	56 (18.9)	296 (8.2)	
Liquid waste disposal	Sink/drain connected to pit	819 (72.7)	308 (27.3)	1127 (31.2)	0.29
	Sink/drain connected to soak pit	489 (76.8)	148 (23.2)	637 (17.6)	
	Disposed directly to open ground	1300 (72.1)	504 (27.9)	1804 (49.9)	
	Other	33 (71.7)	13 (28.3)	46 (1.3)	

^a^Row percentages

^b^Column percentages

^c^Improved water source includes any of piped water into yard, pubic tap/standpipe, protected spring, protected dug well

^d^Improved toilet facility include any of flush/pour flush toilets, septic tank, pit latrine, or unknown destination; ventilated improved pit (VIP) latrines; pit latrines with slabs; and composting toilets; *HEW* health extension worker, *ANC* antenatal care; *HFIAS* Household food insecurity access scale

From the 3,614 sampled children, 250 (5.1%) had SAM (MUAC < 11.5 cm), and 1130 (26.9%) had GAM (MUAC < 12.5 cm). In the bivariate logistic regression analysis, individual-level factors including child age, child sex, Vitamin A supplementation, diarrhea in the last nine months, treatment sought for diarrhea, and fever in the last nine months were significantly associated with child acute malnutrition at *p* < 0.20. Community-level variables such as toilet facility, source of water for drinking, and HFIAS showed significant association with the dependent variable (Table 1). These variables were then entered into the final multilevel multivariable logistic regression model.

There is a considerable variation of childhood acute malnutrition across the zones and districts of Tigray. The lowest and highest prevalence of child acute malnutrition was reported from Mekelle zone (13.3%) and Southeastern zone, respectively (36.7%) (Table 2).

**Table 2:** Zonal variation of acute malnutrition among 6–59 months old children from Tigray, Northern Ethiopia, 2021 (*n* = 3614)

Zone*	Moderate acute malnutrition (MAM), *n* (%)	Severe acute malnutrition (SAM), *n* (%)	Global acute malnutrition (GAM), *n* (%)
Central	197 (22.3)	40 (4.5)	237 (26.8)
Eastern	280 (24.3)	36 (3.1)	316 (27.4)
Mekelle	26 (9.6)	10 (3.7)	36 (13.3)
Northwestern	76 (15.1)	34 (6.7)	101 (21.8)
Southern	104 (23.3)	40 (8.9)	144 (32.2)
Southeastern	106 (29.9)	24 (6.8)	130 (36.7)
Total	789 (21.8)	184 (5.1)	973 (26.9)

*Western zone of Tigray was not included in the study for security reasons

**Fig. 1:**
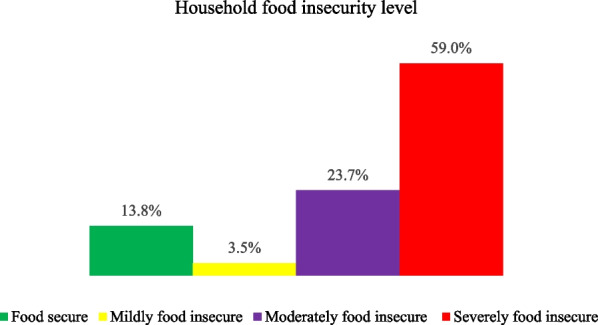
Household food insecurity level of the study participants, Tigray, Northern Ethiopia, 2021 (*n* = 3614)

Regarding household food insecurity, about 2,131 (59%) households were severely food insecure (Fig. 1).

Besides, a one category negative shift in household food insecurity was associated with an 8 mm decrement in the MUAC of a child.

### Multilevel factors associated with child acute malnutrition during the war on Tigray

#### Description of models

In Model 1 (null, empty, or intercept-only model), in which neither individual nor community-level variables were entered, there was a significant variation in the log odds of child acute malnutrition (σ^2^u0 = 0.29, *p* < 0.001, 95%CI: 0.17–0.48). Model 2 represents the significant variation in log odds of acute malnutrition that could be explained by individual variables only (σ^2^u0 = 0.33, *p* < 0.001, 95%CI: 0.20–0.56). About 10% of the association of these variables with childhood acute malnutrition was attributed to clustering effects as shown by an intra-class correlation (ICC) value of 0.1. In Model 3, community-level factors created a significant difference in the log odds of being acutely malnourished (σ^2^u0 = 0.27, *p* < 0.001, 95%CI: 0.16– 0.46) with an ICC value of 0.12. In Model 4 (combined model) both individual and community-level factors significantly affected acute malnutrition (σ^2^u0 = 0.19, *p* < 0.001, 95%CI: 0.14– 0.31) with an ICC value of 0.03. This model was found to be a better fit to the data as explained by its relatively lowest AIC and BIC values (Table 3).

**Table 3:** Multilevel logistic regression model of factors associated with acute malnutrition in 6–59 months age children during the war in Tigray, Northern Ethiopia, 2021 (*n* = 3,614)

Individual and community-level variables		Model 1	Model 2	Model 3	Model 4
		Intercept-only AOR(95% CI)	Individual-level variables AOR(95% CI)	Community-level variables AOR(95% CI)	Multilevel (combined) variables AOR(95% CI)
Child age (months)	6–8 (ref)		1		1
	9–11		1.00(0.82,1.21)		1.03(0.84,1.24)
	12–23		0.58(0.38,0.86)**		0.58(0.38,0.87)**
	24–59		0.12(0.10,0.17)***		0.13(0.10,0.18)***
Child sex	Male (ref)		1		1
	Female		1.24(1.04,1.47)*		1.24(1.05,1.48)*
Supplemented with Vitamin A	Yes (ref)		1		1
	No		1.31(1.02,1.69)*		1.30(1.05,1.65)*
Diarrhea in the last nine months	No (ref)		1		1
	Yes		1.26(1.01,1.57)*		1.22(1.02,1.53)*
Fever	No (ref)		1		1
	Yes		1.25(1.01,1.54)*		1.19(0.96,1.48)
Drinking water source	Improved (ref)			1	1
	Unimproved			1.25(1.05,1.48)**	1.31(1.08,1.58)**
Toilet facility	Improved (ref)			1	1
	Unimproved			1.12(0.94,1.35)	1.24(1.01,1.52)*
HFIAS	Food secure (ref)			1	1
	Mildly food insecure			2.00(1.28,3.12)**	2.36(1.42,3.91)**
	Moderately food insecure			1.10(0.82,1.47)	1.14(0.82,1.58)
	Severely food insecure			1.58(1.22,2.04)***	1.55(1.16,2.07)***
Random error	Community level variance (SE)	0.29(0.03)***	0.33(0.029)***	0.27(0.04)**	0.19(0.02)***
	ICC	0.15	0.10	0.12	0.03
Model fit statistics	AIC	16,354.505	9,213.778	16,423.071	8,322.105
	BIC	16.360.696	9,219.845	16,429.260	8,328.231

Significant at *p < 0.05; ** *p* < 0.01; *** *P* < 0.001

*AOR* Adjusted Odds Ratio, *CI* Confidence Interval, *AIC* Akaike information criterion, *BIC* Bayesian information criterion, *HFIAS* Household food insecurity access scale, *ref* Reference category

#### Individual-level factors

During the multivariable multilevel logistic regression analysis, individual level factors including child age, child sex,, vitamin A supplementation, and diarrhea in the last nine months were found to be significantly (*p* < 0.05) associated with child acute malnutrition in 6–59 months old children from the study communities (Table 3).

Compared to children aged 6–8 months, children in the age range of 24–59 months showed 87% lower (AOR = 0.13, 95% CI: 0.10, 0.18) odds of acute malnutrition. The odds of acute malnutrition were 24% higher (AOR = 1.24, 95% CI: 1.05, 1.48) among female children as compared to their male counterparts. Besides, children who didn’t receive vitamin A supplementation had 30% higher odds (AOR = 1.3, 95%CI: 1.05, 1.65) of acute malnutrition compared to those who did. Moreover, the odds of acute malnutrition were 22% higher (AOR = 1.22, 95%CI: 1.02, 1.53) among children who had diarrhea when compared to their counterparts (Table 3).

#### Community-level factors

Community-level factors including source of water for drinking, toilet facility, and HFIAS were also significantly associated with the dependent variable in the multivariable multilevel logistic regression analysis. The odds of acute malnutrition for those who drink water from unimproved source were 1.3 times higher (AOR = 1.31, 95%CI: 1.05, 1.58) as compared to their counterparts. Children from households with mildly food insecure and severely food insecure household showed more than twice (AOR = 2.36, 95%CI: 1.42, 3.91), and 1.55 times higher odds (AOR = 1.55, 95% CI: 1.16, 2.17) of acute malnutrition, respectively. The log odds of acute malnutrition were 24% higher (AOR = 1.24, 95% CI: 1.01, 1.52) in children from households with unimproved toilet facilities in contrast to those from households with improved toilet facilities (Table 3).

## Discussion

Acute malnutrition (global: 26.9%, moderate: 21.8% and severe: 5.1%) was found to be a serious public health problem among 6–59 months old children from the armed conflict affected settings of Tigray, Northern Ethiopia. Remarkable deterioration of the nutritional status of children was observed compared to the 2.9% and 8.9% prevalence of SAM and GAM before the onset of the armed conflict [18] amounting to a relative increment of 43.1% and 66.9%, respectively. Besides, our findings were pretty consistent with UNICEF’s technical report that revealed a GAM rate of 23.8%—34.3% among children under-five years of age in Tigray during the war corresponding to phase 5 of the Integrated food security Phase Classification (IPC) = ‘famine’ [19]. Moreover, our findings were similar to other studies which reported a high burden of acute malnutrition in armed conflict settings from Somalia, 21% [11], South Sudan, 19% [20], and Nigeria, 19.2% [10]. The high burden of acute malnutrition among 6–59 months old children from Tigray represents an acute situation of recent poor nutrition that might have been caused by inadequate diet or illness exacerbated by the armed conflict waged in Tigray highlighting that acute malnutrition remains a serious risk to the children’s growth and development, as well as to the long-term development of Tigray particularly in terms of poor human health, lost human capital, and decreased economic productivity.

Children in the age category of 24–59 months had 87% lower risk of acute malnutrition compared to children 6–8 months, which is different from a finding reported by a study conducted under normal condition in Bangladesh that showed an increased risk of malnourishment with increasing age [21]. Late introduction of the complementary foods and higher incidence of vaccine preventable diseases could be the potential reasons for the poor nutritional status of the younger children (6–8 months) compared to the older children (24–59 months). In communities affected by conflict, food prices are skyrocketed and the agriculture and market systems are disrupted as evidenced by the high level of household food insecurity in the communities. Hence, mothers would resort to continue with the provision of breastmilk to the young children and delay introduction of complementary foods at six months of age. However, after six months of age, it is difficult for breastfed infants to meet their nutrient needs from human milk alone [22]. Data from Demographic and Health Surveys have shown that malnutrition is higher among children with incomplete vaccination schedules [23]. The under-vaccinated 6–8 months old children in this study did not complete their vaccination schedule and hence are exposed to higher incidence of vaccine preventable infections [24] and eventually poor nutritional status.

Our finding also indicated that children who reported not to have been supplemented with vitamin A had 30% higher odds of acute malnutrition. This is supported by another study from Ethiopia [25]. In agreement with this, taking vitamin A capsule regularly prevented children from vitamin A deficiency in China [26]. Vitamin A plays an essential role in enhancing immune response and hence children affected with Vitamin A deficiency have a tendency to be more affected by infection and malnutrition. Thus, early introduction of children to abundant natural sources of vitamin A or periodic supplementation of vitamin A is suggested to prevent infection and ultimately acute malnutrition [27]. However, in conflict affected communities, the health systems and infrastructures are damaged and essential health services including the periodic supplementation of vitamin A are disrupted which greatly affect the nutritional status of children.

Our study depicted that children whose source of drinking water was unimproved had increased odds of acute malnutrition. In line with this, children who had diarrhea in the last nine months preceding the survey had 22% higher odds of acute malnutrition. Both of this associations indicate that lack of safe water for domestic consumption results in water-borne diseases like that of diarrheal diseases which in turn could result in poor appetite and metabolic and clinical disturbances that lead to poor nutrient utilization and eventually acute malnutrition. This is strongly supported by a comparative study in armed conflict and non-conflict settings of Sudan [13] that stated children of disadvantaged households with low quality of services, such as unsafe drinking water and poor levels of hygiene and sanitation, are more vulnerable to enteric pathogens that cause greater risk of severe episodes of diarrhea highlighting the importance and contribution of symptomatic and asymptomatic infections to child undernutrition.

Our study also found a significant association between unimproved toilet facilities and child acute malnutrition. Unimproved toilet facilities encourage open defecation that in turn creates a conducive environment for diarrheal and other infectious diseases to develop and spread across communities. Other studies conducted in Ethiopia [28] and Bangladesh [29] have reported similar results. Infections are recognized as important causes of poor appetite in children and of metabolic and clinical disturbances that lead to poor nutrient utilization [5]. Possible mechanisms for the symptomatic enteric infections leading to growth faltering include reduced nutrient absorption through lower intestinal contact time during episodes of acute diarrhea, greater nutrient losses from persistent diarrhea or intestinal bleeding, reduced appetite, and diversion of energy and nutrients from growth to the immune system to fight the infection [30, 31]. Moreover, children with asymptomatic environmental enteropathy are believed to have impaired growth through reduced nutrient absorption due to decreased surface area in the small (upper) intestine and elevated intestinal permeability, which increases translocation of antigenic molecules that stimulate the immune system and divert energy from growth. The combined effect of these two processes may impair a young child’s ability to effectively utilize nutrients in the prevailing poor diet for growth and development [32–34]. Notwithstanding these associations, Water, sanitation, and hygiene (WASH) practices and acute malnutrition did not have a cause and effect relationship that is strong enough for developing preventive or curative WASH interventions specifically aimed at dealing with acute malnutrition [35].

In this study, children in food insecure households from armed conflict settings showed higher odds of acute malnutrition compared to those from food secure households. Besides, a one category negative shift in household food insecurity was associated with an 8 mm decrement in the MUAC of a child. This is supported by a recent report by the World Food Program (WFP) that described the food security situation in Tigray as worrisome, with 83% of households being food insecure and more than four out five households consuming inadequate diets [36]. As a result of conflict, the capacity to resist food insecurity was declined among Gazan households [37]. Additionally, due to the conflict-induced food insecurity, children from Burundi and Zimbabwe were significantly affected by chronic malnutrition [38]. Similarly, the armed conflict in Syria has caused more than six million people face severe food insecurity [39]. The impact of armed conflicts on agriculture is through the disruption of farming systems operated by households that reduces outputs of specific staples and harvested areas eventually leading to household food insecurity [40]. The household agricultural production and incomes through extension support, linkages to markets, village savings and loan, and small-scale irrigation schemes are disrupted and hence may have aggravated the household food insecurity level of the target population accounting for the high burden of malnutrition in children from food insecure households. Strong associations between contemporary armed conflict and food insecurity [41] with an aggravating effect on child acute malnutrition [42] have been reported. Furthermore, the inadequate concentrations of macro and micronutrients and/or poor bioavailability in the staple diets can be recognized as an additional dietary limitation putting the children from food insecure households at risk for acute malnutrition.

The main strength of this study is that it used primary data from large sample size that covers wide geographical area. The fact that it used a multilevel analysis also offsets the possible bias that could have been introduced due to clustering. Limitations include the fact that this study, due to its natural design, couldn’t show cause-effect relationship and the possible effect of confounders even though we used multilevel logistic regression analysis, and that some factors like infant and young children feeding practices and household wealth were not assessed.

## Conclusions

Acute malnutrition among 6 to 59 months old children in Tigray is at extremely critical level. Individual-level factors such as child age, child sex, vitamin A supplementation, and diarrhea and community-level factors such as source of drinking water, toilet facility, and household food insecurity were found to be significant factors associated with acute malnutrition among 6–59 months old children. The timely large-scale humanitarian assistance for food, nutrition, WASH, and health services are recommended to prevent the fatal consequences of acute malnutrition in the communities affected by armed conflict. Such services could be provided by international and local non-governmental organizations, not-for profit organizations, and/or other governmental institutions. In the prevailing armed conflict, however, these have been very difficult to achieve. Thus, immediate international intervention is needed.

## Supplementary Information

**Additional file 1.** Dataset used in this study.

## Data Availability

All data generated or analysed during this study are included in this published article [and its Additional file 1].

## Abbreviations

ANC: Antenatal care
AOR: Adjusted odds ratio
CI: Confidence interval
COR: Crude odds ratio
GAM: Global acute malnutrition
HEW: Health extension workers
HFIAS: Household food insecurity access scale
MUAC: Mid upper arm circumference
SAM: Severe acute malnutrition
SD: Standard deviation
WASH: Water, sanitation, and hygiene
WFP: World food program
WHO: World health organization
